# The Action of Medicines in the System, or “On the Mode in Which Therapeutic Agents, Introduced into the Stomach, Produce Their Peculiar Effect on the Animal Economy:” Being the Prize Essay to Which the Medical Society of London Awarded the Fothergillian Gold Medal for 1852

**Published:** 1860-01

**Authors:** 


ART. IV.—*The Action of Medicines in the System*, or "On the Mode in which Therapeutic Agents, introduced into the Stomach produce their peculiar effect on the Animal Economy:" Being the Prize Essay to which the Medical Society of London awarded the Fothergillian Gold Medal for 1852. By Frederic William Headland, M. D., B. A., F. L. S. Licentiate of the Royal College of Physicians, etc., etc. Third edition; revised and enlarged. Philadelphia: Lindsay & Blakiston, 1859.

That a third edition has been demanded, is a sufficient indication of the favor which this admirable essay has met with from the profession. Works like this, upon special subjects, do not, usually, have ready sale; and the demand for a third edition of such a book, shows a much greater proportionate success than four or five, of a book which could be used generally, as a text-book. Publishers well know that text-books are the works to sell; and they are much more anxious to get hold of such, than to publish the most elaborate and learned monograph, which are always uncertain as regards pecuniary success. Our publishers, however, have found it for their interest to republish many foreign monographs, as well as text-books; and the success of some of them shows that though the former do not sell as well as the latter, yet they have not miscalculated on the taste of a great number of the profession, and that many are glad to buy works on special subjects. The mechanical execution of the book before us is admirable, and we doubt, indeed, if it be not equal to the English edition. There is certainly no reason why we should not have our type as clear, and our paper as good as the English; and we venture to say that the English impression of this is little, if any, better than ours. Within a year or two, especially, have we rema ked a great improvement in the mechanical execution of works issued by Lindsay & Blakiston and Blanchard & Lea, bringing them quite up to the English standard. It is certainly a great privilege for us to have reprints as well executed, and at a lower price than the originals.

Of the work before us, it is only necessary to say that it has enjoyed a very high reputation, which is amply merited; and the new edition has been thoroughly revised by the author, who has also somewhat enlarged the scope of the work.

